# Tofacitinib for the treatment of refractory immune checkpoint inhibitor-associated immune nephritis

**DOI:** 10.1093/ckj/sfae127

**Published:** 2024-04-25

**Authors:** Kiran Shivaraj, Amanda Tchakarov, Yanlan Dong, Jamie S Lin

**Affiliations:** Section of Nephrology, Division of Internal Medicine, The University of Texas MD Anderson Cancer Center, Houston, TX, USA; Department of Pathology and Laboratory Medicine, University of Texas Health Science Center McGovern Medical School, Houston, TX, USA; Section of Nephrology, Division of Internal Medicine, The University of Texas MD Anderson Cancer Center, Houston, TX, USA; Section of Nephrology, Division of Internal Medicine, The University of Texas MD Anderson Cancer Center, Houston, TX, USA

**Keywords:** acute interstitial nephritis, immune checkpoint inhibitors, immune nephritis, immune-related adverse events, tofacitinib

## Abstract

Immune checkpoint inhibitor (ICI)-associated immune nephritis or acute interstitial nephritis (AIN) is one of the rare but known complication of ICI therapy. Guidelines recommend treatment of ICI-associated AIN with steroids, then TNF-alpha inhibitor infliximab. However, some cases are refractory to these therapies, potentially due to insufficient cytokine blockade. This is the first case where a 65-year-old female with metastatic lung adenocarcinoma, requiring high maintenance doses of steroids for immune nephritis was treated with tofacitinib, an oral Janus kinase (JAK) inhibitor. Tofacitinib enabled successful steroid tapering and might be a therapy option for refractory immune nephritis.

## BACKGROUND

The inappropriate activation of the Janus kinase/signal transducers and activators of transcription (JAK/STAT) pathway is associated with cancer pathogenesis and autoimmune diseases. Currently, the JAK inhibitor tofacitinib is approved for autoimmune diseases resistant to biologics, including psoriatic arthritis, rheumatoid arthritis, and refractory ulcerative colitis. Given the pivotal role of JAK/STAT signaling in autoimmune pathogenesis, this pathway is also implicated in the development of immune-related adverse events (irAEs) resulting from immune checkpoint inhibitor (ICI) therapy.

Current guidelines for ICI-associated acute interstitial nephritis (AIN) recommend glucocorticoid therapy as the first line, followed by biologic agents such as infliximab. However, there are no clear guidelines for cases refractory to biologic therapy. In this report, we present the first case of a patient with ICI-associated AIN treated with tofacitinib, a JAK inhibitor, following the failure of glucocorticoid and tumor necrosis factor (TNF) blockade to induce renal remission of ICI-associated AIN.

## CASE REPORT

**Figure 1:**
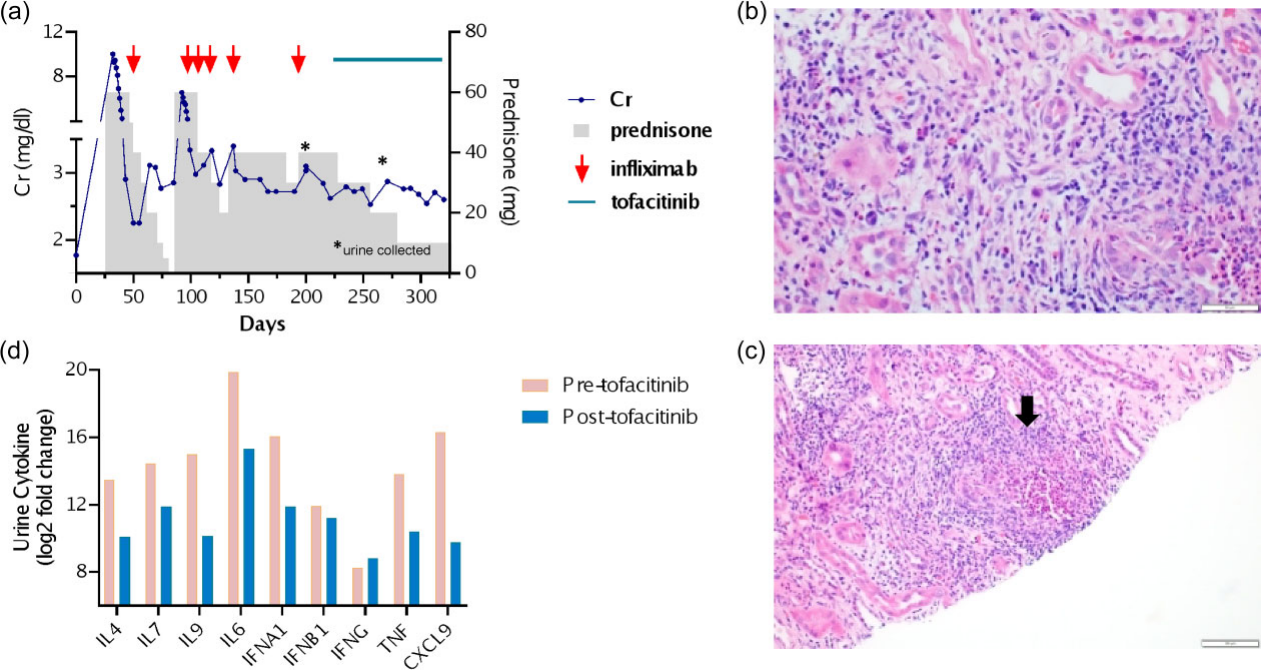
(**a**) Treatment course of a patient with ICI-associated immune nephritis. The patient had an initial response to prednisone (gray columns) and infliximab (5 mg/kg dose, red arrow). Despite extended glucocorticoid therapy and repeated infliximab infusions, the patient required a high maintenance dose of prednisone. The addition of tofacitinib (5 mg daily) enabled prednisone tapering. Urine was collected before and after starting tofacitinib (asterixis). (**b** and **c**) Representative biopsy images. (**b**) Severe tubulointerstitial inflammation with numerous eosinophils and acute tubular injury [hematoxylin and eosin (H&E), 40×]. (**c**) non-necrotizing granuloma (black arrow) (H&E, 20×). (**d**) Cytokines associated with Janus kinase (JAK) signaling were analyzed in a urine specimen collected before starting tofacitinib (pre-tofacitinib, pink columns) and after starting tofacitinib (post-tofacitinib, blue columns). Abbreviations: IL, interleukin; IFN, interferon; A, alpha; B, beta; G, gamma; and CXCL, C-X-C chemokine ligand.

In May 2022, a 65-year-old female with stage IV lung adenocarcinoma with 60% positivity for PD-L1 and pleural and pericardial metastases was hospitalized due to severe acute kidney injury while undergoing treatment with carboplatin, pemetrexed, and pembrolizumab. Her past medical history included history of smoking (quit >10 years ago), hypertension, sleep apnea, and no known history of cardiovascular disease. Laboratory tests indicated an elevation in serum creatinine (Cr) levels from a baseline of 1.6–1.8 mg/dl to 9.9 mg/dl (Fig. 1a) and a serum bicarbonate level of 16 mEq/L (normal 22–29 mEq/L). Her serum sodium and potassium levels were within normal range. Urinalysis (UA) revealed >182 white blood cells per high-power field (WBC/HPF) and numerous urine eosinophils. The urine protein to creatinine ratio (UPCR) was 1.07 g/g. Renal ultrasound ruled out hydronephrosis. A kidney biopsy confirmed AIN with granuloma formation along with acute tubular injury (Fig. 1b and c). The patient received treatment with prednisone 60 mg once daily tapered over 6 weeks and a single infusion of infliximab at a dose of 5 mg/kg. Fortunately, she did not require renal replacement therapy. Post-therapy, she experienced partial renal recovery with a Cr level of 2.8 mg/dl, UA 8 WBC/HPF, and UPCR 0.37 g/g.

Two weeks later, laboratory studies revealed a deterioration in kidney function (Cr 6.56 mg/dl, UA with 26–50 WBC/HPF, negative urine eosinophils, and UPCR 0.94 g/g) attributed to the relapse of ICI-associated AIN. Prednisone with infliximab therapy was restarted. Despite an additional 4 months of prednisone therapy and five cycles of infliximab, the patient remained unresponsive to biologic treatment, requiring a high maintenance prednisone dosage of 40 mg to maintain stable kidney function (Cr 2.7–2.9 mg/dl). In November 2022, tofacitinib at 5 mg daily was introduced to her prednisone regimen. Subsequently, the patient's kidney function stabilized (Cr 2.5–2.7 mg/dl) and by January 2023 she successfully tapered her prednisone to 10 mg once daily. Repeat UA was negative for WBC, urine eosinophils, and UPCR 0.09 g/g. To investigate whether the improvement in renal function was associated with tofacitinib therapy, urine specimens collected before tofacitinib therapy in October 2022 and after tofacitinib therapy in December 2022 were examined [4]. Urine cytokine analysis by Nucleic Acid Linked Immuno-Sandwich Assay (NULISA^TM^) revealed a reduction in inflammatory urine cytokines levels associated with JAK inhibition (Fig. 1d).

In late January, progression of her underlying cancer was discovered. Tofacitinib was discontinued, and prednisone 10 mg was continued, while chemotherapy with Taxotere was initiated. Two months later, the patient passed away due to complications arising from her underlying malignancy.

## DISCUSSION

To our knowledge, this is the first report of tofacitinib use for ICI-associated AIN. In this patient who required high maintenance prednisone therapy, tofacitinib treatment stabilized the patient's renal function and she was able to successfully taper to a low prednisone dose. Although we did not attempt to completely taper off the glucocorticoids, this case, coupled with the success of tofacitinib therapy for other irAEs—colitis, myocarditis, and hepatitis—underscores its potential as a promising target for further investigation in patients experiencing refractory irAEs [1–3].

Cells driving irAEs such as in ICI-associated AIN include cytotoxic CD8^+^ T cells that produce substantial amounts of cytokines such as TNF, interferons, and interleukins [4]. Despite the therapeutic achievements of biologics, targeting a single cytokine may not fully eliminate irAEs. The JAK/STAT signaling pathway induces the expression of >50 cytokines and growth factors [5]. Consequently, small molecules aimed at JAKs are emerging as a treatment for autoimmune and inflammatory diseases.

Tofacitinib functions as primarily a JAK1/JAK3 inhibitor. Tofacitinib, by inhibition of JAK3, inhibits cytokines IL-2, IL-4, IL-7, IL-9, IL-15, and IL-21; and by JAK1 inhibition, inhibits IL-6, type 1 IFNs, and IFN-gamma [5]. Metabolized by CYP3A4, it has a half-life of 3 hours, with ∼30% undergoing renal excretion. For patients with moderate to severe kidney dysfunction, once-daily dosing is recommended [5]. Notably JAK inhibitors offer advantages such as oral administration and rapid onset of action within days to weeks. Baseline laboratory studies and routine monitoring should include a complete blood count and differential, complete metabolic, and lipid panel. Patients should also be screened for latent tuberculosis and hepatitis. Owing to elevated risk of thromboembolic disease, caution should be used in those with cardiovascular disease [5].

An increased risk of cancer has been noted with long-term usage of tofacitinib. Given the relatively short duration of tofacitinib therapy received by our patient, we consider it less likely that tofacitinib contributed to cancer progression, particularly in the context of prolonged high-dose steroid treatment. The FDA recommends tofacitinib usage for rheumatoid arthritis or ulcerative colitis in patients refractory to biologics. We chose to initiate tofacitinib in our patient based on these recommendations. Further research is warranted to explore the effects of JAK inhibitors in the setting of ICI therapy and immunotoxicity.

The clinical application of JAK inhibitors offers a therapeutic avenue for patients with autoimmune disease proven resistant to biologics. A parallel clinical demand arises for individuals experiencing refractory irAEs such as ICI-associated AIN. This report seeks to establish the foundation for future therapeutic approaches, highlighting tofacitinib as a potential treatment for refractory immune nephritis.

## PATIENT CONSENT

The patient provided written informed consent.

## Data Availability

The data underlying this article are available in the article.
